# Teleworking as a work modality: reflections from institutional analysis

**DOI:** 10.47626/1679-4435-2021-890

**Published:** 2023-11-24

**Authors:** Brenda Reis Chaves, Flávia Lúcia da Silva Colares, Ester Mendes da Silva, Daniel Vannucci Dóbies, Carla Aparecida Spagnol

**Affiliations:** 1 Escola de Enfermagem, Universidade Federal de Minas Gerais (UFMG), Belo Horizonte, MG, Brazil; 2 Faculdade de Ciências Médicas, Universidade Estadual de Campinas, Campinas, SP, Brazil; 3 Departamento de Enfermagem Aplicada, Escola de Enfermagem, UFMG, Belo Horizonte, MG, Brazil

**Keywords:** teleworking, COVID-19, occupational health, personnel management, working conditions, institutional analysis, teletrabalho, COVID-19, saúde do trabalhador, administração de recursos humanos, condições de trabalho, análise institucional

## Abstract

This opinion article addresses teleworking, which has gained momentum in Brazil due to the COVID-19 pandemic. The discussions arose from a course in the Professional Master’s Degree in Health Services Management at a nursing school in the state of Minas Gerais, Brazil. The authors raised teleworking-related questions, which are not only affecting their lives, but also the lives of workers in general, causing problems socially, economically, and related to workers’ health. The reflections were drawn up using some concepts from institutional analysis. This article aimed to analyze teleworking as an analyzer of work in the context of the COVID-19 pandemic. As teleworking rapidly took off, the benefits were envisioned, but the potential detriments of this type of work were not considered. As a result, many professionals working from home began to work in unsuitable conditions, lacking the necessary infrastructure and support to perform their activities, such as ergonomic furniture, equipment, materials, internet access, technical assistance, training, and support. However, teleworking should not be overlooked, as many workers have identified with this type of work and many organizations have benefited from it becoming institutionalized. However, we cannot remain “numb,” waiting for some miraculous reversion to pre-pandemic conditions, in order to organize the new type of “normal” in the world of work in a less alienated way.

## Abstract (pt)

O presente artigo de reflexão aborda a modalidade de trabalho home office, que se intensificou no Brasil a partir do contexto da pandemia da covid-19. As reflexões foram originárias de discussões realizadas em uma disciplina do Curso de Mestrado Profissional em Gestão de Serviços de Saúde, de uma Escola de Enfermagem do estado de Minas Gerais. Os autores problematizaram questões relacionadas ao home office, que estão interferindo em suas vidas, mas, também, na vida dos trabalhadores de modo geral, ocasionando problemas sociais, econômicos e relativos à saúde do trabalhador. As reflexões foram elaboradas em articulação com alguns conceitos da Análise Institucional. O objetivo é analisar o home office considerando-o como analisador do trabalho no contexto da pandemia da covid-19. A partir da célere instalação do trabalho remoto, idealizou-se os benefícios e não foram considerados os possíveis prejuízos dessa modalidade de trabalho. Assim, diversos profissionais em home office passaram a trabalhar em condições inadequadas, sem a infraestrutura necessária e o suporte para a realização das atividades, como a disponibilização de mobiliário ergonômico, equipamentos, materiais, acesso à internet, assistência técnica, capacitações e acompanhamentos. Entretanto, não se pode desprezar a alternativa do home office, pois diversos trabalhadores se identificaram com essa modalidade de trabalho e muitas organizações se beneficiam com a sua institucionalização. Ao mesmo tempo, não se pode ficar “entorpecido”, à espera de um milagroso regresso à realidade pré-pandêmica, para organizar de forma menos alienada o novo tipo de “normalidade” no mundo do trabalho.

## INTRODUCTION

The first cases of infection due to the novel coronavirus (SARS-CoV-2) occurred in the Chinese city of Wuhan in early December 2019.^1^ The virus spread extensively and rapidly, causing the disease called COVID-19, which the World Health Organization (WHO) declared a pandemic on March 11, 2020, and Brazil declared a state of public calamity on the 20th of March.^2,3^

Therefore, the COVID-19 pandemic has triggered abrupt changes in the lives of the entire world population. In the absence of effective treatments and vaccines against the disease, social distancing became an essential preventive measure.^4^ As a result, several public and private services were forced to introduce, within a very short period of time and where applicable, that their employees worked from home. This decision was intended to ensure productive activities could be continued, while avoiding social contact that could spread the disease.

It is estimated that around 81% of the world’s workforce has been affected by changes in their workplace.^5^ In Brazil, figures from the Pesquisa Nacional por Amostra de Domicílios (PNAD-COVID-19, Brazil National Household Sample Survey) revealed that 8.3 million workers were working from home by the first week of September 2020.^6^

Shortly after the pandemic was declared, Brazil enacted Medida Provisória (MP, Provisional Measure) 927/2020, which allowed employers to change from on-site to teleworking, working remotely, or any other type of remote work, without the need for a mutual agreement in a labor contract. This MP provided that, if the worker did not have the necessary and adequate infrastructure and technology devices to work remotely, the organization could provide these devices on a commodatum arrangement, for example, and pay for infrastructure services, which would not constitute a salary.^7^

On April 1, 2020, the Brazil Administration published MP 936/2020 to supplement MP 927/2020, proposing labor alternatives to cope with the public calamity resulting from the COVID-19 pandemic, in order to reduce the effects on employment and income in formal jobs, while also reducing the social impacts of the public calamity. This new MP covered temporary suspension of the labor contracts, reduced working hours, and proportional salary, in addition to the payment of Beneficio Emergencial de Preservação do Emprego e da Renda (Emergency Employment and Income Maintenance Program).^8^

It should be noted that MP 927/2020 expired and Congress failed to pass it into law. However, some measures, such as reduced working hours and suspending contracts, set out in MP 936/2020, were extended and passed into Law No. 14,020/2020. This law establishes the Emergency Employment and Income Maintenance Program and provides for additional relief measures for the state of public calamity and public health emergency, which is of global importance, due to SARS-CoV-2.^9^

In this context, in which labor laws have emerged as countermeasures to the crisis caused by the pandemic, one of the forms of teleworking that has gained popularity is home office. In this format, workers work from home, or anywhere outside the employer’s physical premises, which is a type of teleworking regulated by an internal company policy. Teleworking can also be referred to as the use of information and communication technologies.^10,11^

This scenario has allowed most organizations to promote various advantages of teleworking, such as: reduced commuting time, possible productivity gains, increased worker motivation, better work/life balance, more flexibility, and greater control over activity schedules. At the beginning of the pandemic, these promises made teleworking an “object of desire” for many workers because it seemed to be an opportunity to reconcile work and social distancing, maintaining the usual work tasks with the benefit of less exposure to SARS-CoV-2.

These conditions have allowed teleworking to expand and intensify. At the same time, there have been challenges and disadvantages to this type of teleworking: increased household costs and sedentary lifestyles, inevitable communication problems (poor signal), and a combination of work and home tasks. In addition, there has been increased ergonomic risk, owing to the poor infrastructure for a working environment within the home, which can lead to musculoskeletal disorders. These problems combined certainly make it difficult to perform some activities.^12^

In addition to this scenario, there is also psychological suffering, such as stress, anxiety, isolation, among others. These anxieties undoubtedly affect productivity, health, and occupational well-being.^13^

As teleworking has been increasingly common in recent years and can make working conditions more precarious, there are some precautions and strategies that should be adopted to create a favorable environment for the development of these activities, such as: ensuring the continuity of work, providing infrastructure and support to perform tasks, setting up a long-term teleworking policy, time management, and reducing working hours.^14^

From these considerations, which portray an acceleration and possible trend towards greater growth of teleworking in Brazilian society, this opinion article was drawn up to discuss this type of work.

These reflections arose from discussions held in the classroom and from a final assignment for an elective course taught as part of the Professional Master’s Degree in Health Services Management at a nursing school in the state of Minas Gerais. This course was taught in the form of emergency remote teaching, from November 30, 2020, to March 8, 2021, and had a 45-hour duration.

This article’s authors include three who were enrolled in this course, two nurses, and one doctor, who work in health services located in Belo Horizonte, Minas Gerais. These health personnel do not work directly in providing care to users, but rather in auditing, continuing education, and occupational health, which has allowed them to do most of their work remotely during the pandemic.

This circumstance was an invitation encouraging the authors to debate issues related to teleworking, which has been interfering significantly in their lives, as well as in the lives of all workers, causing problems socially, economically and related to workers’ health.

These reflections were drawn up using some concepts from the theoretical and methodological framework of institutional analysis (IA), and to further discuss and strengthen the collective production of this article, the coordinator of the subject and a PhD student from another university participated as authors, having been invited to teach a class on the concept of analyzer, which is one of the central concepts of IA.

In light of the above, this article aimed to analyze teleworking as an analyzer of work in the context of the COVID-19 pandemic.

### TELEWORKING AS A WORK ANALYZER: IMPLICATIONS FOR PROFESSIONAL PRACTICE

In the midst of the social event of COVID-19 pandemic, teleworking can be approached as an analyzer of social relations in general, and labor relations in particular. Considering teleworking as an analyzer indicates that it can provoke analyses that denaturalize the ongoing social process, as is proposed in the IA theoretical and methodological framework created by René Lourau and Georges Lapassade.

Denaturalizing events and social arrangements, analyzing the set of normative and institutional crossings, enables subjects to see themselves as involved and not subjected to what happens to them, which expands the possibilities of acting, retreating, peeking and/or denying in a detached way.

This detached way of looking at the world of work allows workers, even in a turbulent context, to make their analyses. One of them is that teleworking is nothing new, since many professionals have been working remotely long before the COVID-19 pandemic. However, the pandemic has “forced” many organizations to embrace it to maintain their productivity. As a result, something completely inconceivable for the vast majority of workers just a few years ago has become something that many consider to be their “new normal.”

It could even be said that working from home was a dream for many people, as it would allow for more flexible working hours and new daily configurations, and in a pandemic setting, less exposure to the risk of getting and spreading COVID-19 and other diseases. In addition, the stress and risks of commuting would be over for the time being. The time that used to be wasted in traffic could be time well spent with family and loved ones, resting, and having fun. The hours spent preparing to leave home and commute would become precious minutes of extra sleep.

For those living on their own, teleworking could improve concentration, as interruptions from other people would be less frequent or nonexistent. The environment would be more welcoming and pleasant; everyday arguments about, for example, the “temperature of the air conditioning” or the “window open or closed” would no longer exist. It would be possible to eat healthily, more calmly, and more enjoyably. Working from home would also give workers the option of resolving domestic issues themselves and accessing services that are only available during business hours.

Teleworking would also be capable of providing something that used to be totally impossible, such as being in other towns while still being able to work, ie, being able to work from anywhere. Therefore, teleworking would not mean “working from home” at all, as it would allow goals to be met remotely, from anywhere on the planet, simply by having a computer, a cell phone, and internet access.

*A priori*, teleworking would provide a number of benefits and several of these gains have actually been achieved. However, practically speaking, this has not proved to be entirely true. Despite being the desire of many professionals, the shift from the workplace to homes, amid the pandemic, has occurred quickly and abruptly, affecting both the work process and people’s lives. In this sense, teleworking has been revealed as an analyzer in the world of work.

According to Lapassade, analyzers have analytical material that leads people to analyze its implications.^15^ Lourau adds that this work is not limited to a revelation, but to a process of displacement and deconstruction when he states that “the institution has the power to objectify us, to thingify us in statutes and roles. The analyzer ‘de-objectifies’: it undoes statutes and roles, it restores our subjectivity. [...] the institution has the power to materialize in apparently neutral and universal forms, at everyone’s service, economic and political forces that dominate us, pretending to help and defend us. The analyzer dematerializes the forms of oppression, revealing the forces that hide in them, and combats any material forms.”^16^

Thus, analyzing the implications can help some professionals not to be deluded by the “object of desire” (teleworking). They can, for example, analyze how their home spaces have changed abruptly, with dining tables turned into desks, and living rooms (bedrooms, hallways and other “nooks and crannies” of the house) turned into improvised offices and meeting rooms. As a result, workers began to use their personal computers and their internet access for their work tasks, while the lighting and furnishings were inadequate and uncomfortable to use for long hours. The immediate demand to maintain production meant that these professionals either failed to analyze the various signs of precariousness at work or were unable to curb this process.

In general, many workers have had to change their homes to provide a pleasant and comfortable environment for their work. However, some organizations failed to provide financial and material resources, leaving it up to the professionals to make the financial investment needed to adapt their environment. In this case, the economic conditions became favorable for the companies and, on the other hand, unfavorable for the workers, who had to bear the costs of a “makeshift office.”^17^

The *Instituto Brasileiro de Geografia e Estatística* (IBGE, Brazilian Institute of Geography and Statistics) reinforces that, for many organizations, the way to contain the spread of SARS-CoV-2, the way to “isolate” their workers and the faster means to perform certain jobs from home have been accompanied by a deterioration in working conditions.^6^

Therefore, embracing a “new working environment” called home office, using an Anglicism typical of contemporary times and corporate language, promises status and novelty, yet conceals the effects of the ongoing precariousness of work and the heterogeneity of this practice across Brazil. There are very different conditions when working from home, depending on the architecture and structure of the property, the composition and relationship of the family, the neighborhood, the quality of internet access, among other factors. Unless personal and collective singularities are considered when it comes to working in a home environment, we are left with the simplistic idea that everything can be adapted over time, based on a few practical adjustments and personal willingness.

This alleged sophistication of the term home office needs to be desacralized so that conditions can be created for analysis of singularities and micro-political confrontations. For example, a flexible working day can be confused with a surplus of hours and/or inappropriate periods of work (late at night, on days off), and managers benefiting from this supposed flexibility, often believe that they have the right to call on workers outside of their working hours. The issue of time really needs to be analyzed, as it directly affects workers’ health. In addition, the idea of a better diet at home can lead to excessive consumption of sweets and irregular mealtimes. Similarly, breaks may not be taken.

Another analysis, which could not be left out of this discussion, is the working conditions of workers who share their time with taking care of family members, especially children, who started doing their schoolwork at home during the pandemic. In this context, work is affected in many ways, as tasks can become fragmented, meetings can be interrupted and silence becomes impossible when children demand more attention. Even children are analyzers of this situation, as they are less bound to social norms. In this case, they display how hard it is to adapt within a short time and how much our society devalues and/or pays little attention to children’s education.

It should also be recognized in this situation that the burden of childcare and caring for family members in general falls more heavily on women, which is another critical point of teleworking for certain people. Reconciling work and family care, which teleworking facilitates at first, also brings enormous risks of emotional and work burdens, which need to be included in the analysis as a whole, rather than simply being naturalized.

In addition, discussions need to be held about interprofessional relationships, given that digital platforms now represent “meeting rooms, corridors and coffee corners,” which can lead to a loss of emotional ties, due to the lack of greater multiplicity in face-to-face interaction with other people. After all, we are social and emotional beings, and removing the intricacies of interpersonal relationships can negatively affect our emotional condition and sense of belonging.

The sudden rise of teleworking as the pandemic hits highlights just how much this was a historic boom moment, when changes occurred with no planning, no transitions, and no care for people. Certainly, previous teleworking experiences and the telecommunication technology available are two factors that have provided the necessary materiality to follow the course of this accelerated institutionalization.

However, other characteristics of current times have contributed to this happening: the naturalization and generalization of productivism. The fact that productive responsibilities fall to individuals in a society marked by neoliberal rationality, combined with the growing threat of job losses at a critical point in the pandemic, has increased compliance with this orientation for teleworking.

More generally, we can see that the enormous social turmoil the pandemic has caused has led to teleworking becoming institutionalized as a new working model for a number of professionals: administrators, teachers, salespeople, artists, journalists, some people in the health sector, among others. Clearly, not all professionals, not even those in the categories listed, have migrated to teleworking, especially as it is impossible to perform certain work tasks remotely. Nonetheless, teleworking has brought about countless changes in the world of work.

The singularities of teleworking performed by workers in the emergence of the pandemic must therefore be considered, along with the extent to which and how this model of work leads to changes in professional practices, so as not to trivialize its consequences for workers, employers, and society as a whole.

## FINAL CONSIDERATIONS

The question of teleworking as an analyzer draws a line of social connections to be observed, including: the agility to gain productivity at the expense of loss of quality standards, subsequent labor and administrative reforms that withdraw workers’ rights in Brazil, economic-profit motivations overlapping with any others, the advancing use of technology in professional practices and social relations in general, among others.

From this perspective, the COVID-19 pandemic has interrupted, but also created and accelerated various practices. Nevertheless, the prompt installation of teleworking has shown a desire to maintain productivity, using all available resources. The benefits were idealized and no consideration to potential losses was considered.

As a result, many professionals working remotely continue to work in unsuitable conditions. The adaptations needed to the home space, the acquisition/updating of devices, materials, better internet plans, have only been possible for a few privileged people. Moreover, workers are not able to keep up with, learn from, and deal with all these new practices. All this leads to countless inequalities.

Therefore, teleworking should include demands and needs that can improve working conditions, such as: provision of ergonomic furniture, devices, materials, internet access, technical assistance, capacity building and support. These measures can prevent missing an opportunity to implement something that could even be beneficial to employees and employers, as well as society as a whole and the environment.

It should be noted that the gains of teleworking cannot be undervalued, as many workers have certainly identified themselves with this working model, and many organizations have benefited from it being institutionalized. In any case, we cannot remain “numb,” waiting for a miraculous reversion to pre-pandemic conditions (something we do not know when, or even if, will happen) in order to organize the new type of “normality” in the working environment in a less alienated way.
